# The bidirectional brain–cochlea axis: a scaffold for neurologic disease-associated hearing loss

**DOI:** 10.1093/braincomms/fcae403

**Published:** 2024-11-09

**Authors:** Arianna Di Stadio, Elliot M Frohman, Daniela Messineo, Michael J Brenner, Evanthia Bernitsas

**Affiliations:** GF Ingrassia Department, Otolaryngology Unit, University of Catania, Catania, Italy; Neuroimmunology Laboratory of Professor Lawrence Steinman, Stanford University School of Medicine, Palo Alto, CA, USA; Dipartimento di Scienze Radiologiche, Oncologiche ed Anatomo Patologiche, University La Sapienza, Rome, Italy; Department of Otolaryngology—Head & Neck Surgery, University of Michigan Medical School, Ann Arbor, MI, USA; Department of Neurology, Creighton University, Ohama, NE, USA

## Abstract

This scientific commentary refers to ‘Beyond the cochlea: exploring the multifaceted nature of hearing loss in primary mitochondrial diseases’, by Koohi *et al*. (https://doi.org/10.1093/braincomms/fcae374).

**This scientific commentary refers to ‘Beyond the cochlea: exploring the multifaceted nature of hearing loss in primary mitochondrial diseases’, by Koohi *et al*. (https://doi.org/10.1093/braincomms/fcae374).**

In their recent article in *Brain Communications*, Koohi *et al*.^1^ explored hearing disturbances in patients with mitochondrial disease. The authors analysing this population concluded such disturbances could arise from both cochlear damage and involvement of central auditory pathways. Moreover, they emphasized the role of auditory processing deficits in causing altered hearing.^1^ Mitochondrial disease causes bioenergetic deficits that affect multiple organs. This multi-systemic involvement is evident in the auditory pathways, involving peripheral mechanisms (in the cochlea) and central mechanisms (in the brainstem and auditory cortex).

Koohi *et al*.^1^ have combined traditional audiologic assessments with cognitive testing, for a more comprehensive understanding of how mitochondrial disease affects the auditory system. Pure-tone audiometry and distortion-product otoacoustic emissions confirmed inner ear dysfunction, while altered auditory brainstem responses indicated involvement of central auditory pathways. These findings provide critical insights into how mitochondrial dysfunction affects both the periphery and central processing centres of hearing. Additionally, the authors documented diminished cognitive performance, suggesting that mitochondrial disease exerts widespread effects across multiple systems.

Authors’ results support the concept that impairments in mitochondrial oxydative phosphorylation and electron transport chain affect bioenergetic supply and demand in mitochondrial disease. Mitochondrial dysmetabolism reduces the production of ATP and increases the production of reactive oxygen species (ROS), resulting in toxicity and cellular apoptosis in both cochlear hair cells and neurons in the superior auditory pathways.^1,2^ A high concentration of ROS into the brain triggers the overexpression of disease modifying microglia (DAM), activated cells that contribute to neuroinflammation, neurodegeneration and cognitive impairment.^3^ Although Koohi *et al*.^1^ did not perform biochemical analyses, their clinical findings—a decline of cognitive ability—might support this causal mechanism.

DAM, induced by excess ROS, contribute to neuroinflammation, which in turn leads to further ROS production. Elevated neurofilament light chain (Nf-L) levels are considered a marker of brain neuroinflammation^4^; therefore, their concentration could reflect the severity of brain damage. Recent studies in adolescents with mitochondrial disease have identified a direct correlation between Nf-L levels in CSF and the severity of brain degeneration.^5^ This biomarker can also be found in the CSF of patients affected by other conditions, such as multiple sclerosis^6^ and Alzheimer’s disease^7^; the high concentrations of Nf-L correlate with the progression^6^ and severity^7^ of these conditions, confirming the prognostic value of Nf-L as a marker of neuroinflammation.

Koohi *et al*.^1^ focus on mitochondrial disease, but the findings may have broader implications as similar auditory pathway disruptions are observed in other neurodegenerative conditions such as multiple sclerosis and Alzheimer’s disease. In these disorders, auditory dysfunction often mirrors the same central and peripheral mechanisms implicated in mitochondrial disease, suggesting a shared pathophysiological basis involving bioenergetic deficits and neuroinflammation.^8^ The presence of both cochlear and central auditory involvement in such diseases underscores the need for a more integrated approach to understanding hearing loss in neurological contexts where the brain–cochlea axis plays a pivotal role. The question arises, ‘Could the hearing impairment in these disorders reflect a brain–cochlea axis rather than an incidental concomitant condition associated with neurological disease?’

Recent research has provided evidence that injury mechanisms in mitochondrial disease can originate in either the peripheral or central nervous system, and the injury can spread bidirectionally. Soluble mediators such as ROS and immune factors traverse anatomical structures, including the cochlear aqueduct (CA) and internal auditory canal (IAC), to spread between the inner ear and the brain. Lessons learned from paediatric neurological populations reveal that many genetic disorders culminate in sensorineural hearing loss due to the exchange of infectious and inflammatory mediators between the subarachnoid space and the inner ear. In some cases, inner ear infections can seed the CSF, further illustrating the bidirectional interaction of the inner ear and central nervous system. This bidirectional scaffolding provides a potential pathway for disease processes to migrate between the periphery and central nervous system.

This migration could explain why some patients with mitochondrial disease present with such diverse neurological symptoms, including auditory deficits, cognitive impairments and motor dysfunctions. CSF can carry different inflammatory elements^5,7,8^ like ROS, L-NF and lactate through the CA (Fig. 1) and IAC^9^ (Fig. 2). They are all markers of neuroinflammation; indeed, their elevated concentrations are correlated to neuronal degeneration.^5-7^ The role of ROS in damaging the inner ear is well known,^2^ so direct damage caused by high concentrations of these elements migrating from the brain into the cochlea can be a plausible cause of hearing loss.

**Figure 1:**
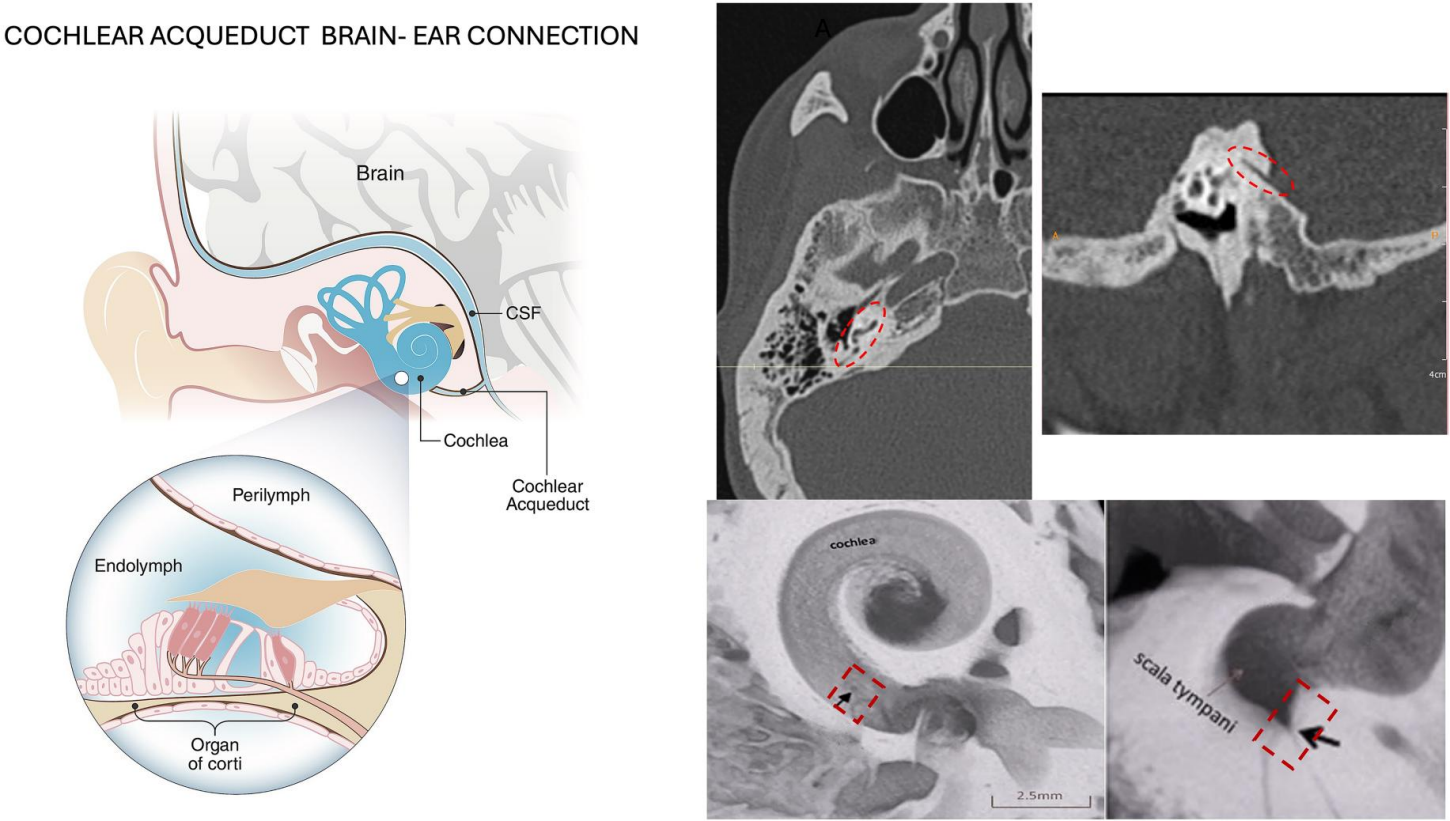
**Fluid exchanges between brain and inner ear through the cochlear aqueduct**. The drawing shows the fluid exchanges between brain and inner ear through the cochlear aqueduct (left side). On the right side, the cochlear aqueduct (interrupted circle in the image) is visible in CT scan (axial and sagittal view) with microscopic view of its position in the cochlea (black arrow), modified from Li Z, Shi D, Li H, *et al*. Micro-CT study of the human cochlear aqueduct. *Surgical and Radiologic Anatomy* 2018;40(6):713-720. doi: 10.1007/s00276-018-2020-6 by permission of Springer Nature.

**Figure 2:**
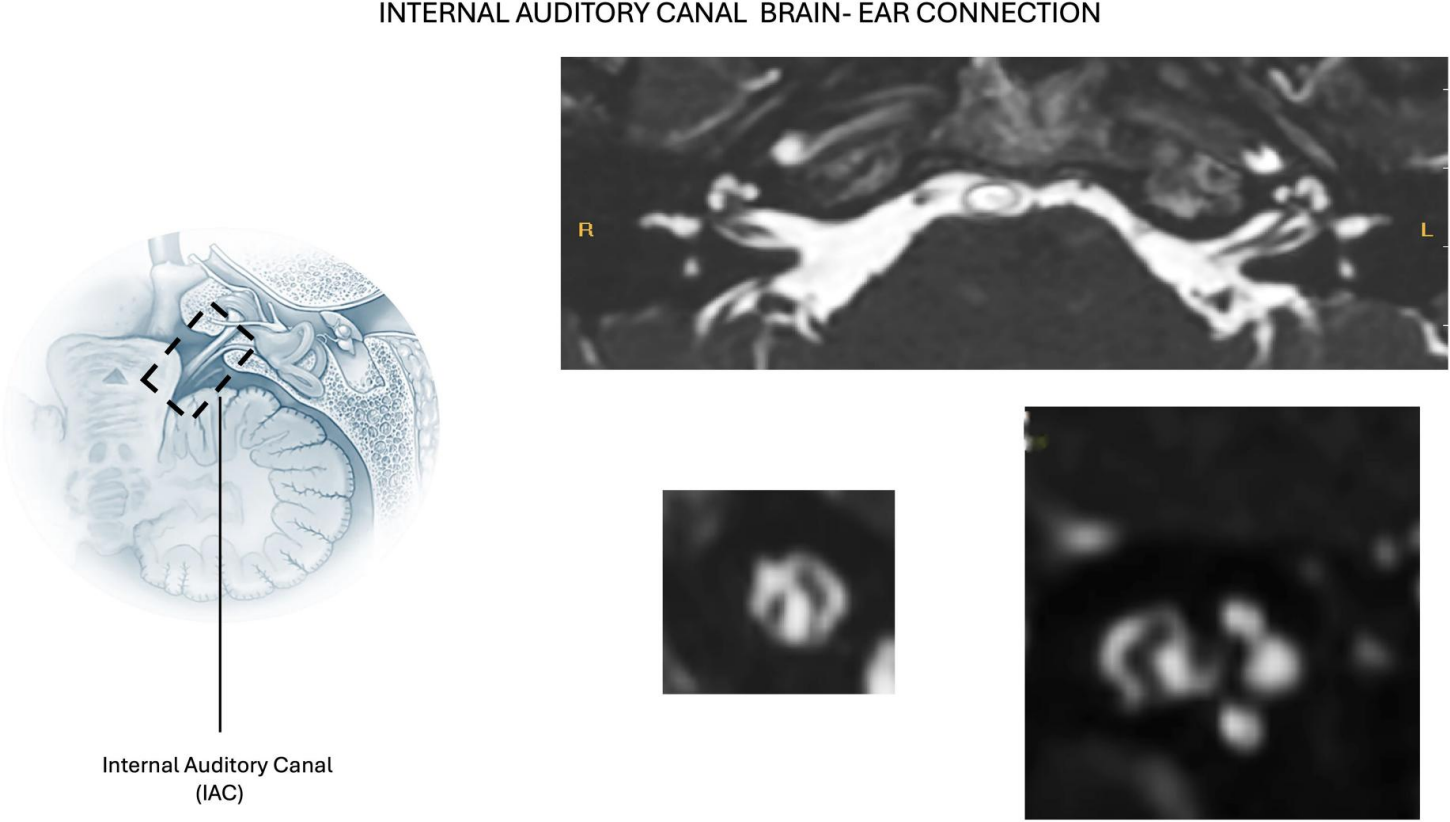
**Fluid exchanges between brain and inner ear through the internal auditory canal**. The drawing shows the fluid exchanges between brain and inner ear through the internal auditory canal (IAC) (left side). On the right, axial, coronal and sagittal views of the IAC with its fluid content are shown.

In the setting of mitochondrial dysfunction, impaired oxidative phosphorylation causes inefficient production of ATP, redox imbalance and excess production of ROS that contribute to elevate lactate levels in the CSF. High concentrations of lactate in the CSF^5^ that reaches the cochlea might damage the inner ear,^10^ explaining the finding of worse auditory thresholds in presence of excess of lactate.^10^ Lactate causes vasodilation of inner ear vessel and hyper-perfusion of the stria vascularis.^10^ A rapid rise in lactate levels, like what occurs in a focal haemorrhagic stroke, may stress the stria vascularis and lead to cochlear injury with the typical down-sloping pattern of hearing loss on audiogram. Moderate fluctuations of lactate into the CSF, as observed in relapsing-remitting multiple sclerosis)^6^, may cause cyclic perturbations of blood flow that cause progressive damage of the stria vascularis.

Neuroinflammatory and neurodegenerative disorders, like mitochondrial disease, are characterized by shared immune-oxidative alterations. Oxidative stress has been largely studied, and the role of ROS as contributor to disease progression is well known. Recent advances link molecular injury effectors to sources such as microglia in the central nervous system, which are responsible for producing ROS in response to disease stimuli. These discoveries underscore the importance of targeting oxidative stress and immune responses in developing new treatments for hearing loss associated with mitochondrial disease and other neurodegenerative disorders.

Because lactate concentration is correlated with brain injury,^5^ monitoring auditory function in mitochondrial disease might afford a window into brain damage. As shown by Koohi *et al*.,^1^ there is a correlation between mitochondrial disease genotype and severity of mitochondrial disease and hearing loss. Their evidence supports the notion that findings on peripheral auditory tests might indirectly represent brain findings. Although the inner ear might be the first anatomic target to suffer from neuroinflammation along the bidirectional brain–cochlea axis, the presence of peripheral damage often signals concomitant deficits in auditory processing and other higher brain functions. Given the complexity of hearing deficits in mitochondrial disease and other neurological conditions, collaboration between neurology and otolaryngology is essential to foster a multidisciplinary approach to identify and address the underlying mechanisms.

Future studies on mitochondrial disease can achieve more comprehensive disease characterization by combining physiological tests and cognitive assessments with brain MRI and testing for biomarkers of CSF neuroinflammation (e.g. Nf-L and lactate measurement).

Such investigations could clarify the interplay of neuroinflammation/neurodegeneration with hearing functions and discern at what level (peripheral, central or both) interactions occur. It would be also interesting to expand the investigation on blood microRNA.^2^ The expression of these elements is correlated to the inner damage and can identify which structures of the inner ear are affected (hair cells, spiral ganglia, or both?).^2^ Measurement of blood circulating microRNA could also clarify if the difficulties in word discrimination are caused by a deficit in the superior auditory cortex or by the severe loss of spiral ganglions—the peripheral structure deputed to word discrimination.

In summary, bidirectional brain–cochlea axis represents a critical scaffolding for understanding the intersection of auditory and neurological dysfunctions in conditions like mitochondrial disease. Determining whether hearing loss is related to the underlying neurological condition or is an independent finding is crucial for guiding appropriate treatments. Auditory dysfunction might serve as an early indicator of broader neurological degeneration. Furthermore, hearing loss associated with neurodegenerative disorders may not respond to therapies targeted at the periphery. For example, amplification or cochlear implantation is less likely to confer benefit to patients with central auditory processing deficits. Personalized auditory rehabilitation can integrate auditory assessments with neurobiological insights for more effective, personalized assessment and management approaches.

## Data Availability

Data are available under reasonable request to the corresponding author.
